# Seasonal effect on semen availability and quality of racing pigeon in Thailand

**DOI:** 10.14202/vetworld.2021.1459-1464

**Published:** 2021-06-07

**Authors:** Suwarak Wannaratana, Em-on Olanratmanee, Kuekaroon Charoenmuang, Thanvarath Boriharnthanawuth, Banpatee Tangtrongwanich, Thanawan Jongpattana, Yanita Sukhor, Arrita Kongthip, Thanida Sananmuang

**Affiliations:** Faculty of Veterinary Medicine, Rajamangala University of Technology Tawan-Ok, Chonburi, Thailand

**Keywords:** humidity, photoperiod, pigeon semen quality, temperature, Thailand

## Abstract

**Background and Aim:**

Seasonal variations among geographical regions could influence pigeon semen quality differently. This study aimed to determine the seasonal effect on semen availability and quality of racing pigeons in Thailand to understand and improve breeding management in the country.

**Materials and Methods:**

Semen was collected from six fertile captive pigeons once a week during summer (March-June), monsoon (July-October), and winter (November-February) during 2019-2020. The success rate of semen collection and semen quality was determined in each season – by which changes in average temperature, humidity, and photoperiod were obtained.

**Results:**

Comparable success rates of semen collection were acquired among different seasons, while varied semen qualities were revealed. The percentages of total motility and progressive motility score of sperm were significantly lowest in summer (66.35±3.40 and 3.88±0.15, respectively) compared to monsoon (85.45±2.91 and 4.67±0.10, respectively) and winter (79.29±1.96 and 4.37±0.10, respectively), while its concentration (×10^9^ sperm/mL) and outputs (×10^6^ sperm) were significantly highest in winter (7.62±0.54 and 91.44±10.83, respectively) compared to summer (4.23±0.41 and 48.45±6.35, respectively) and monsoon (3.57±0.30 and 51.45±7.21, respectively). Besides, semen samples collected from birds housing at an average temperature of <29.5°C demonstrated better sperm motility sperm concentration and total sperm counts than those from at a higher temperature.

**Conclusion:**

Winter was regarded as the best season contributing the best semen quality, while summer was the worst. Due to the fluctuation of temperature during summer and winter, the seasonal temperature was implied as the major factor contributing to changes in sperm quality of racing pigeons in Thailand.

## Introduction

Pigeon racing has been regarded as one of the most popular bird sports worldwide, including in Thailand [1,2]. To obtain pigeons with greater racing potentials, their breeding strategy is an important factor. Despite its annual breeding, only a few offspring of genetically exceptional pigeons is successfully bred. Striving to exploit genetic potential from such prestigious pigeons introduced assisted reproductive technologies (ARTs) in racing pigeons [3]. Among well-recognized ARTs, artificial insemination and semen cryopreservation are the most promising technologies in avian species [4]. To establish such technology, semen evaluation is a conventional tool for monitoring the reproductive performance of male birds [5-8] and improving breeding strategy for racing purposes in pigeons [9,10]. Seasonal variation’s effects on semen quality have been previously reported in several avian species, including pigeons [11-14]. Such influences were majorly contributed to temperature, and photoperiod varied among seasons. Interestingly, several studies even indicated varied seasonal effects among different geographic regions – which possess unique climate characteristics. It should also be noted that continuous climate change driven by human activities could also complicate the seasonal effect on semen quality. However, recent studies even manifested the influence of climate change on bird reproduction worldwide [15,16]. Alternative breeding performance of several avian species living in temperate zones has been reported, including changing breeding time, changing clutch size, the timing of nesting, reproductive success, and offspring sex ratios due to climatic change [17-20].

In temperate zone countries, such as Spain, the success rate of semen collection in amazons and macaws is highest in spring, while no semen collection is possible in autumn and winter [11]. On the contrary, pigeons in the Netherlands – which are located in a temperate zone but with a maritime climate, the success collection rate was highest in winter, while the highest volume was obtained in autumn [12]. Thus, these studies supported the contribution of geographical factors on semen availability and quality of the same climate zone. Thailand is a country located in a tropical zone. Seasons and climate of the tropical region are unique from those presented in the temperate zone – by which narrower ranges of temperature throughout seasons are common. The tropical region was well-recognized for its hot and humid climate, which contradicts those observed in the temperate zone. In several mammals and cocks in Thailand, the qualities of their semen were superior in the winter season [21-24]. Interestingly, Thai native cock seemed to be tolerated on fluctuated climatic changes among seasons [24]. Such acknowledgment implied the possible unique seasonal effect on semen characteristics of various species, including pigeons in Thailand.

Due to very limited knowledge about the seasonal effects on racing pigeons in Thailand, this study aimed to gain insight into the effect of seasonal variation on semen availability and quality acquired from racing pigeons. By acquiring data of climate parameters – temperature, humidity, and photoperiod, their effects on racing pigeon’s semen in Thailand were first explored in this study.

## Materials and Methods

### Ethical approval

Experimental procedures were approved by Rajamangala University of Technology Tawan-Ok Animal Ethics Committee (RMUTTO-ACUC-2-2019-003), and care was taken to minimize the number of animals used.

### Study period and location

This study was carried out from March 2019 to February 2020 at the Faculty of Veterinary Medicine, Rajamangala University of Technology Tawan-Ok in Chonburi province, the eastern part of Thailand.

### Animals

A total number of six fertile racing adult male pigeons aged 1-2 years weighing 400-450 g were used. They were kept individually in metal cages and placed in the opened house exposed to natural environmental conditions. The birds were fed with 10 g of complete feed twice daily and water available *ad libitum*.

### Experimental design

The semen was collected from six fertile captive pigeons once a week during summer (March-June), monsoon (July-October), and winter (November-February) from 2019 to 2020. The success rate of semen collection, semen quality, and meteorological data – average temperature, humidity, and photoperiod acquired among seasons was compared.

### Semen collection and evaluation

Semen was collected from birds once a week by lumbosacral massaging and gentle squeezing at the base of the cloaca performed by the same collector throughout the study. The collected semen was diluted in LRS at 25-fold dilutions and was immediately evaluated for their quality [25]. The macroscopic evaluation included the determination of semen volume, pH, and color. The microscopic evaluation included determination of total sperm motility, progressive sperm motility, sperm viability, sperm concentration, total sperm count, and sperm morphology.

#### Semen volume and pH

The ejaculate volume was determined using a calibrated displacement pipette, while the pH was determined using pH indicator strips (Riedel-De-Haёn AG, Germany). Each of the evaluation techniques was performed by the same evaluator for standardization.

#### Sperm motility evaluation

The percentage of total motile sperm and progressive motility was evaluated under a light microscope at 100× for 10 fields. Progressive motility of the sperm was graded on a 5-point score (0 indicates no motility, 1 indicates non-progressive motility, 2 indicates slow progressive motility, 3 indicates side to side movement accompanied by slow progressive motility, 4 indicates faster progressive motility, and 5 indicates very fast progressive motility) [3].

#### Sperm viability and morphology evaluation

Semen samples stained with eosin-nigrosin dye were assessed for sperm viability and morphology. The proportions of live (eosin-impermeable) and dead (eosin-permeable) sperm in a sample were assessed based on 200 sperm counts. For morphology evaluation, 300 sperm counts acquired from each sample were assessed for normal, amorphous head, bent head, macrocephalic head, acrosomal defect, loosed head, abnormal midpiece, proximal droplet, coiled tail, bent tail, distal droplet, loosed tail, and double tail sperms under a light microscope at 1000×.

#### Sperm concentration

Sperm concentration was assessed by diluting 10 μL of sperm suspension in 990 μL of formal saline (100-fold dilution). The diluted sperm suspension was transferred to a counting chamber (Boeco, Germany) and the sperm concentration was then evaluated under a light microscope at 400×.

### Meteorological data

The data of average temperature (°C), average humidity (%), and average photoperiod (hours/day) were acquired from the Thai Astronomical Society and Thai Meteorological Department during 2019-2020. According to Thai Meteorological Department data, Chonburi Province was located at 13.22 °N, 100.59 °E, classified as a tropical climate zone. All seasons were categorized as follows: (i) Summer: March-June; (ii) monsoon: July-October; and (iii) winter: November-February.

### Statistical analysis

Normal distribution of the data (p<0.05) was confirmed using the D’Agostino and Pearson omnibus normality test. Student’s t-test was utilized to determine differences between two sample groups of interest, while one-way analysis of variance with Tukey’s test for *post hoc* analysis was utilized to compare three sample groups (p<0.05).

## Results

### Changes in temperature, humidity, and photoperiod among seasons

Significant differences in average temperature, humidity, and photoperiod among the seasons were observed. Summer had the highest average temperature and longest photoperiod, while monsoon had the highest humidity compared to the others (p<0.05) (Table-1).

**Table-1:** Variables of average temperature, humidity, and photoperiod among seasons are expressed as mean±SE.

Variables	Seasons		

	Summer	Monsoon	Winter
Average temperature (^o^C)	30.70±0.11^a^	29.42±0.11^b^	28.47±0.14^c^
Average humidity (%)	72.98±0.45^b^	78.13±0.55^a^	68.39±0.74^c^
Average photoperiod (hours/day)	12.28±0.02^a^	12.12±0.03^b^	11.30±0.01^c^

Different superscripts within the same row indicated significant differences (p<0.05). SE=Standard error

### Variation of semen availability and quality among seasons

The acquired success rate of semen collection (approximately 80-95%), semen volume (ranging between 10 and 13 mL), and sperm viability (approximately 84-86%) was not different among the seasons. The pH of semen was lowest in summer (p<0.05). Interestingly, marked decreases of total sperm motility and progressive sperm motility, along with marked increases of abnormal sperms (macrocephalic head, acrosomal defect, and abnormal midpiece), were manifested in summer (p<0.05). On the contrary, sperm concentration and total sperm counts were highest in winter (p<0.05). Moreover, the percentage of normal sperm was significantly highest in monsoon than in the other seasons (p<0.05) (Table-2).

**Table-2:** Semen quality compared among the seasons in Thailand. Data are expressed as mean±SE.

Parameters	Summer	Monsoon	Winter
Total motility (%)	66.35±3.40^b^	85.45±2.91^a^	79.29±1.96^a^
Progressive motility (score 0-5)	3.88±0.15^b^	4.67±0.10^a^	4.37±0.10^a^
Sperm concentration (×10^9^/mL)	4.23±0.41^b^	3.57±0.30^b^	7.62±0.54^a^
Total sperm counts (×10^6^)	48.45±6.35^b^	51.45±7.21^b^	91.44±10.83^a^
Morphologically normal sperm (%)	74.25±1.59^b^	89.14±1.47^a^	74.42±1.80^b^
Morphologically abnormal sperm (%)			
Amorphous head (%)	4.75±0.68^a^	2.48±0.78^a^	4.41±0.68^a^
Bent head (%)	2.33±0.27^a^	1.96±0.28^a^	2.99 ±0.35^a^
Macrocephalic head (%)	1.64±0.15^a^	0.43±0.09^b^	0.68±0.11^b^
Acrosomal defect (%)	1.62±0.23^a^	0.55±0.14^b^	1.20±0.24^ab^
Loosed head (%)	1.76±0.28^a^	1.53±0.34^a^	0.33±0.11^b^
Abnormal midpiece (%)	1.36±0.17^a^	0.35±0.21^b^	0.84±0.20^ab^
Proximal droplet (%)	0.10±0.04^a^	0.02±0.02^a^	0.11±0.06^a^
Coiled tail (%)	0.54±0.15^a^	0.42±0.23^a^	0.80±0.29^a^
Bent tail (%)	5.64±1.01^a^	0.16±0.06^b^	4.63±0.94^a^
Distal droplet (%)	0.08±0.03^a^	0.01±0.01^a^	0.05±0.03^a^
Loosed tail (%)	5.89±0.85^b^	2.92±1.01^b^	9.51±1.09^a^
Double tail (%)	0.04±0.02^a^	0^a^	0.02±0.02^a^

Different superscripts within the same row indicated significant difference (p<0.05). SE=Standard error

### Effect of average temperature, humidity, and photoperiod on semen quality

Since environmental variables temperature, humidity, and photoperiod varied among seasons, their effects on semen quality were evaluated. Considering mean environmental variables 1 week before semen collection, semen samples collected from birds housing at an average temperature <29.5°C demonstrated better sperm motility, sperm concentration, and total sperm counts than those acquired at a higher temperature. Average humidity greater than 72.5% and photoperiod longer than 12 h/day also contributed to decreases in sperm concentration and total sperm counts (Table-3).

**Table-3:** Characteristics of pigeon semen categorized according to the average temperature, humidity, and photoperiod calculated at 1 week before semen collection. Data were expressed as mean±SE.

Parameter	Average temperature 1 week before semen collection		Average humidity 1 week before semen collection		Average photoperiod 1 week before semen collection	
		
	<29.5°C	>29.5°C	<72.5%	>72.5%	<12 h	>12 h
Total motility (%)	79.12±1.78^a^	70.90±3.46^b^	77.57±2.37	74.08±2.55	79.39±1.81	71.86±3.09
Progressive motility (score 0-5)	4.43±0.08^a^	4.03±0.15^b^	4.27±0.12	4.12±0.11	4.42±0.09	4.09±0.13
Viability (%)	87.49±0.70	84.98±1.51	87.92±0.84	85.62±1.09	87.99±0.73	84.97±1.32
Sperm concentration (×10^9^/mL)	6.62±0.47^a^	4.21±0.36^b^	7.60±0.63^a^	4.60±0.33^b^	7.38±0.50^a^	3.99±0.32^b^
Total sperm counts (×10^6^)	78.89±8.72^a^	52.59±6.04^b^	85.94±12.26^a^	58.25±5.71^b^	87.89±9.83^a^	49.72±5.39^b^

Different superscripts within the same row indicated significant difference (p<0.05). SE=Standard error

## Discussion

Unique seasonal effects on racing pigeon semen quality in Thailand were demonstrated in this study – by which the worst and best semen were obtained in summer and winter, accordingly. Supporting the notification about varied factors provided by different regional climates and environments, this study suggested winter as the optimal season for pigeon semen collection in Thailand – which was different from those previously reported in India [3] and the Netherlands [12].

In this study, the success rate of semen collection from racing pigeons was not significantly different among seasons (80-95%). Such notification was different from those previously reported in the temperate zone – by which significant fluctuation of success rate varied through different seasons [12]. Seasonal effects on some semen characteristics acquired in this study were similar to those previously reported in India [3], which is located in the tropical zone like Thailand. Our result reported the highest semen volume during the monsoon season. As expected, such results contradicted those previously reported in the Netherlands – by which both semen volume and viability were greatest in autumn. The lowest percentages of total sperm motility and progressive motility, and the highest percentages of abnormal sperms during summer (p<0.05) demonstrated in this study were also similar to those previously reported in India [3] and the Netherlands [12]. Even though most semen quality characteristics in this study were similar to those reported in India, sperm concentration and total sperm counts acquired from Thai pigeons were highest in winter (p<0.05), while the best results were presented during monsoon in India (p<0.05) [3].

Although Thailand and India are both located in a tropical zone, this study strongly suggested that their differences in geographic influence could integrate with their climatic factors resulting in unique seasonal effects of each country. Different climate and environmental factors, such as ambient temperature, humidity, and photoperiod, were well-recognized for their variations among the seasons. During the time of this study, summer had the highest average temperature (30.70±0.11°C) and longest photoperiod (12.28±0.02 h) than the other seasons (p<0.05). Monsoon had the highest average humidity (78.13±0.55%) than the other seasons (p<0.05). By evaluating changes in these climatic factors on semen quality, the results strongly implied the negative effects of high ambient temperature (>29.5°C) on sperm motility, sperm concentration, and total sperm count (p<0.05). Moreover, sperm concentration and total sperm count were also negatively affected by high humidity (>72.5%) and long photoperiod (>12 h/day) (p<0.05). Concordant with the seasonal effect’s results, the increases of these climate factors were significant during summer seasons.

The effect of heat stress on decreasing sperm quality has been widely reported [26]. In a rooster, heat stress (>30°C) compromises semen output, sperm viability, and motility [27,28]. Heat stress effect on sperm quality was also reported in pigeons – by which, sperm motility decreased at an ambient temperature greater than 28°C closed to 29.5°C, as reported in the current study [12]. To the best of our knowledge, the negative effect of high humidity on sperm in Thailand was only reported in Duroc boar [21]. Our study was among the first reports that imply this negative effect on pigeons.

There is little information about the photoperiod effect on male reproductive performance in pigeons than other avian species. Optimal photoperiod was reported in turkeys [29,30] and broiler breeder males [31,32]. In turkeys, the recommend photoperiod was 9.5-10.5 h/day [29]. In broiler breeders, the photoperiod of >13 h/day contributes to decreased sperm concentration [31] and plasma testosterone concentration once broilers are exposed to long photoperiod 12-22 h/day [32]. These results suggested similar negative effects on other avian species. We also suspected these negative effects in racing pigeons used in this study; thus, further study is required to exclude all other interfering factors that might integrate their effects along with the photoperiod (especially the high ambient temperatures).

## Conclusion

To the best of our knowledge, this study first demonstrates the seasonal effects on the semen quality of racing pigeons in Thailand. Winter was regarded as the best season contributing the best semen quality, while summer was the worst. Annual variation of temperature, humidity, and photoperiod was suggested to cause this contrast – by which hot weather was suspected the major factor that adversely affected the sperm quality.

## Authors’ Contributions

SW and TS: Planned the study design, analyzed data, and drafted the manuscript. EO, KC, TB, BT, TJ, YS, and AK: Performed semen collection and evaluation. All authors read and approved the final manuscript.
